# *In Vitro* Maturation and *In Vivo* Integration and Function of an Engineered Cell-Seeded Disc-like Angle Ply Structure (DAPS) for Total Disc Arthroplasty

**DOI:** 10.1038/s41598-017-15887-4

**Published:** 2017-11-17

**Authors:** J. T. Martin, S. E. Gullbrand, D. H. Kim, K. Ikuta, C. G. Pfeifer, B. G. Ashinsky, L. J. Smith, D. M. Elliott, H. E. Smith, R. L. Mauck

**Affiliations:** 1 Translational Musculoskeletal Research Center, Corporal Michael Crescenz VA Medical Center, Philadelphia, PA USA; 2 Department of Orthopaedic Surgery, University of Pennsylvania, Philadelphia, PA USA; 3 Department of Mechanical Engineering and Applied Mechanics, University of Pennsylvania, Philadelphia, PA USA; 4 Department of Neurosurgery, University of Pennsylvania, Philadelphia, PA USA; 5 Department of Biomedical Engineering, University of Delaware, Newark, DE USA; 6 Department of Bioengineering, University of Pennsylvania, Philadelphia, PA USA

## Abstract

Total disc replacement with an engineered substitute is a promising avenue for treating advanced intervertebral disc disease. Toward this goal, we developed cell-seeded disc-like angle ply structures (DAPS) and showed through *in vitro* studies that these constructs mature to match native disc composition, structure, and function with long-term culture. We then evaluated DAPS performance in an *in vivo* rat model of total disc replacement; over 5 weeks *in vivo*, DAPS maintained their structure, prevented intervertebral bony fusion, and matched native disc mechanical function at physiologic loads *in situ*. However, DAPS rapidly lost proteoglycan post-implantation and did not integrate into adjacent vertebrae. To address this, we modified the design to include polymer endplates to interface the DAPS with adjacent vertebrae, and showed that this modification mitigated *in vivo* proteoglycan loss while maintaining mechanical function and promoting integration. Together, these data demonstrate that cell-seeded engineered discs can replicate many characteristics of the native disc and are a viable option for total disc arthroplasty.

## Introduction

The United States spends an estimated $560 to $635 billion per year on chronic pain conditions^1^ and 70% of these expenditures can be attributed to primary care, hospital, and emergency room visits and pharmaceuticals to treat back pain^1^. Lumbar intervertebral disc degeneration is a natural consequence of aging and, as deficiency in disc function is closely tied to degeneration of its component tissues, degeneration has been implicated as a causative factor in back pain^2–4^. Current surgical strategies for treating symptomatic disc degeneration, such as spinal fusion and total disc arthroplasty, do not restore native joint mechanics and are associated with downstream complications. For example, intervertebral fusion (approximately 400,000 cases per year in the United States^5^) limits mobility and may accelerate degeneration in adjacent discs^6^. Clinical outcomes following traditional arthroplasty with metal-on-plastic prosthetics have been variable and depend on spinal region; particularly, in the lumbar region, the long-term benefit of arthroplasty in comparison to fusion procedures has not been demonstrated^7,8^.

Given the limitations of fusion and arthroplasty, there is intense interest in regenerative solutions to treat disc disease. A number of therapeutic strategies (for each stage of degeneration) are in development to preserve the intervertebral joint. Early in the degenerative process, interventions with cells, growth factors, or other pharmaceutical therapies may maintain disc function by preventing further loss of tissue structure^9–11^. For the treatment of end-stage disc disease, however, where tissue deterioration is advanced and likely irreversible, a more aggressive approach will be necessary. In these circumstances, a biologic replacement disc may be required, where the entirety of the diseased native disc is removed and replaced.

To achieve this goal, we developed engineered constructs that replicate the hierarchical structure and function of the native disc. These constructs are comprised of a multi-lamellar nanofibrous scaffold with fiber alignment that matches the annulus fibrosus (AF) and a hydrogel core that replicates the nucleus pulposus (NP). Previously, we showed that multilayer AF constructs support tissue development when seeded with mesenchymal stem cells (MSCs) and match native mechanical properties^12^. We also showed that a hydrogel NP promotes a chondrogenic phenotype in both MSCs^13^ and NP cells^14^ and drives NP cells to express NP phenotypic markers^14^. When combined, the AF and NP subunits form disc-like angle ply structures (DAPS), which also mature compositionally *in vitro* ^15^, illustrating their potential for total disc replacement. We also developed an *in vivo* rat caudal spine (or tail) model using with an external fixation system to provide a stable orthotopic site for DAPS implantation^16^. Preliminary results demonstrated that acellular DAPS were biocompatible and maintained lamellar structure over time^16,17^. To further this line of inquiry, the objective of this study was to evaluate the long-term performance of cell-seeded DAPS *in vivo*.

We hypothesized that DAPS *in vivo* integration potential would be related to their *in vitro* growth rate^18^, i.e. that immature (rapidly growing) DAPS integrate better into native tissues than mature (stable) DAPS. To test this hypothesis, we established the *in vitro* growth trajectory of cell-seeded DAPS over a 15-week period and then implanted DAPS after pre-culture durations representing immature or mature growth states. We found that DAPS reached or exceeded many compositional and functional benchmarks *in vitro*. However, after *in vivo* implantation, DAPS shifted from PG-rich to collagen-rich after 5 weeks, a phenomenon independent of pre-culture duration. Furthermore, DAPS did not integrate into adjacent vertebrae. In subsequent studies, these deficiencies were overcome by including engineered endplates (eDAPS) to act as an interface between the DAPS and adjacent vertebrae. This attenuated the shift in NP phenotype, promoted integration into the adjacent vertebral bodies, and enabled function under physiologic loads, replicating the functions of the native intervertebral disc. Taken together, these data confirm the ability of engineered intervertebral discs to re-establish many aspects of the native disc and support the continued translation of whole-disc tissue engineering technologies.

## Results

### DAPS *In Vitro* Growth Trajectory

**Figure 1:**
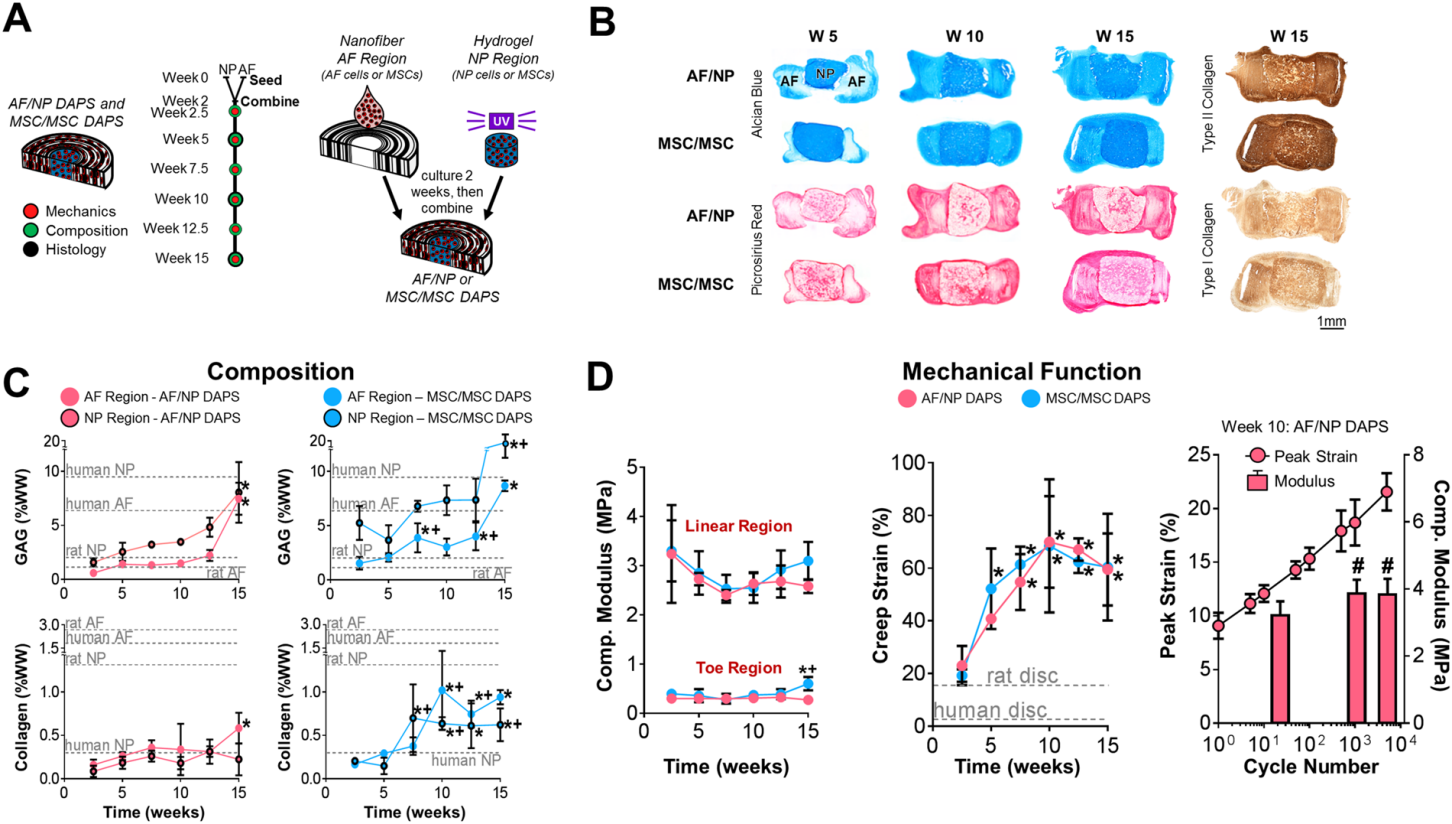
DAPS *In Vitro* Growth Trajectory. **(A)** *Study design:* Engineered intervertebral disc analogs (disc-like angle ply structures, DAPS) composed of a nanofibrous AF and a hydrogel NP, were seeded with either AF and NP cells or MSCs and cultured in chemically defined media with TGF-β3 separately for 2 weeks, and then combined. The *in vitro* maturation of DAPS was evaluated mechanically, compositionally, and histologically at regular intervals over the course of 15 weeks. **(B)** *Alcian blue, picrosirius red, type I and type II collagen staining:* DAPS matured compositionally in terms of proteoglycan (Alcian blue) and collagen (picrosirius red) deposition over this time period, and stained strongly for type II collagen after 15 weeks. **(C)** *Composition*: Histological findings were confirmed by measurements of GAG and collagen content, which exceeded or were within range of native tissue values. GAG content consistently increased over 15 weeks, while collagen content increased early and then reached a plateau. Types I and II collagen-staining indicated a chondrogenic phenotype at 15 weeks. Native values are from^34,54^. (*p < 0.05 vs week. 2.5; + p < 0.05 vs. AF/NP at corresponding time point) **(D)** *Mechanical Function* *:* DAPS demonstrated a nonlinear stress/strain response in compression, and elastic mechanical properties (moduli) that were stable over 15 weeks. Viscoelastic properties (creep strain) became more dominant over the 15-week period, stabilizing by 10 weeks. In both cases, there were no differences between AF/NP and MSC/MSC DAPS, and mechanical properties were within range of native tissue. Native values are from^34,54^. (*p < 0.05 vs. week 2.5; + p < 0.05 vs. AF/NP at corresponding time point). AF/NP DAPS were also tested to determine their fatigue behavior in compression after 10 weeks of culture. The peak strain asymptotically plateaued with increasing cycle number (plotted on a log scale), indicating stiffening of the construct. This was confirmed by measurements of the linear region compressive modulus, which increased from cycle 20 to cycle 1000 (#p < 0.1 vs. cycle 20).

Two versions of DAPS constructs were evaluated for long-term *in vitro* compositional and functional maturation: AF/NP DAPS (AF region seeded with bovine AF cells, NP region with bovine NP cells) and MSC/MSC DAPS (AF region seeded with bovine MSCs, NP region with bovine MSCs). Both construct types were cultured in chemically defined media with the chondrogenic factor TGF-β3 for 15 weeks [Fig. 1A], maturing compositionally with time [Fig. 1B] and increasing in size [Fig. S1]. In the AF region, picrosirius red staining (collagen) and Alcian blue staining (proteoglycan, PG) began at the AF boundaries at 5 weeks and progressed into deeper regions of the AF, with strong positive staining by 10 weeks and reaching peak intensity at 15 weeks. [Fig. 1B]. PG was distributed diffusely through the AF at 15 weeks, while collagen was localized to the interlamellar spaces. In the NP region, both collagen and PG-positive staining increased with time, with collagen staining concentrated at the center of the NP and PG staining evenly distributed throughout the NP at 15 weeks. Immunostaining for type I and type II collagens indicated an NP-like extracellular matrix rich in type II collagen.

*In vitro* maturation was confirmed by glycosaminoglycan (GAG) and collagen content measurements [Fig. 1C]. GAG content increased over time for both AF/NP DAPS (p < 0.05; AF region: 2.5 vs. 15 weeks; NP region: 2.5 vs. 15 weeks) and MSC/MSC DAPS (p < 0.05; AF region: 2.5 weeks vs. 7.5, 12.5, 15 weeks; NP region: 2.5 vs. 15 weeks). Furthermore, GAG production in MSC/MSC DAPS was greater than that of AF/NP DAPS (p < 0.05; AF region at 7.5, 12.5 weeks; NP region at 15 weeks). By 15 weeks, GAG content of both DAPS types reached or exceeded human/rat AF and NP values. With time in culture, collagen content also increased for both AF/NP DAPS (p < 0.05; AF region: 2.5 vs. 15 weeks) and MSC/MSC DAPS (p < 0.05; AF region: 2.5 vs. 10, 12.5, 15 weeks; NP region: 2.5 vs. 7.5, 10, 12.5, 15 weeks). Collagen production in MSC/MSC DAPS was greater than that of AF/NP DAPS (p < 0.05; AF region at 10, 12.5 weeks; NP region at 7.5, 10, 15 weeks). For both DAPS types, collagen content reached or exceeded native human benchmarks in the NP region, but not the AF region.

The mechanical properties of DAPS were evaluated to assess their capacity for *in vivo* function. For both DAPS types, elastic (time-***in***dependent) mechanical properties were largely unchanged over 15 weeks *in vitro* [Fig. 1D]. Conversely, the viscoelastic (time-***de***pendent, related to water influx/efflux) effects became increasingly dominant. Specifically, the toe and linear region compressive moduli (elastic properties) changed minimally over 15 weeks. In contrast, there was a significant increase in creep strain (a viscoelastic property) over the first 10 weeks that plateaued between 10 and 15 weeks for both groups (p < 0.05; AF/NP DAPS: 2.5 vs. 7.5, 10, 12.5, 15 weeks, MSC/MSC DAPS: 2.5 vs. 5, 7.5, 10, 12.5, 15 weeks). Similarly, the early and late responses to creep load became stronger with time in culture, as evidenced by viscoelastic model parameters [Fig. S1]. Thus, DAPS were increasingly viscous with time, progressing from a cell-seeded biomaterial to a hydrated, engineered tissue.

Fatigue tests were performed to assess DAPS function in a dynamically loaded environment. After 10 weeks of *in vitro* culture, AF/NP DAPS supported 5,000 loading cycles, gradually stiffening with increasing cycle number, with no indication of load-induced failure. Peak strain followed an asymptotic trend and linear modulus increased with cycle number [Fig. 1D] (p < 0.05; Linear region compressive modulus: 20 vs. 1000, 5000 cycles).

### Trajectory-based *In Vivo* DAPS Implantation

**Figure 2:**
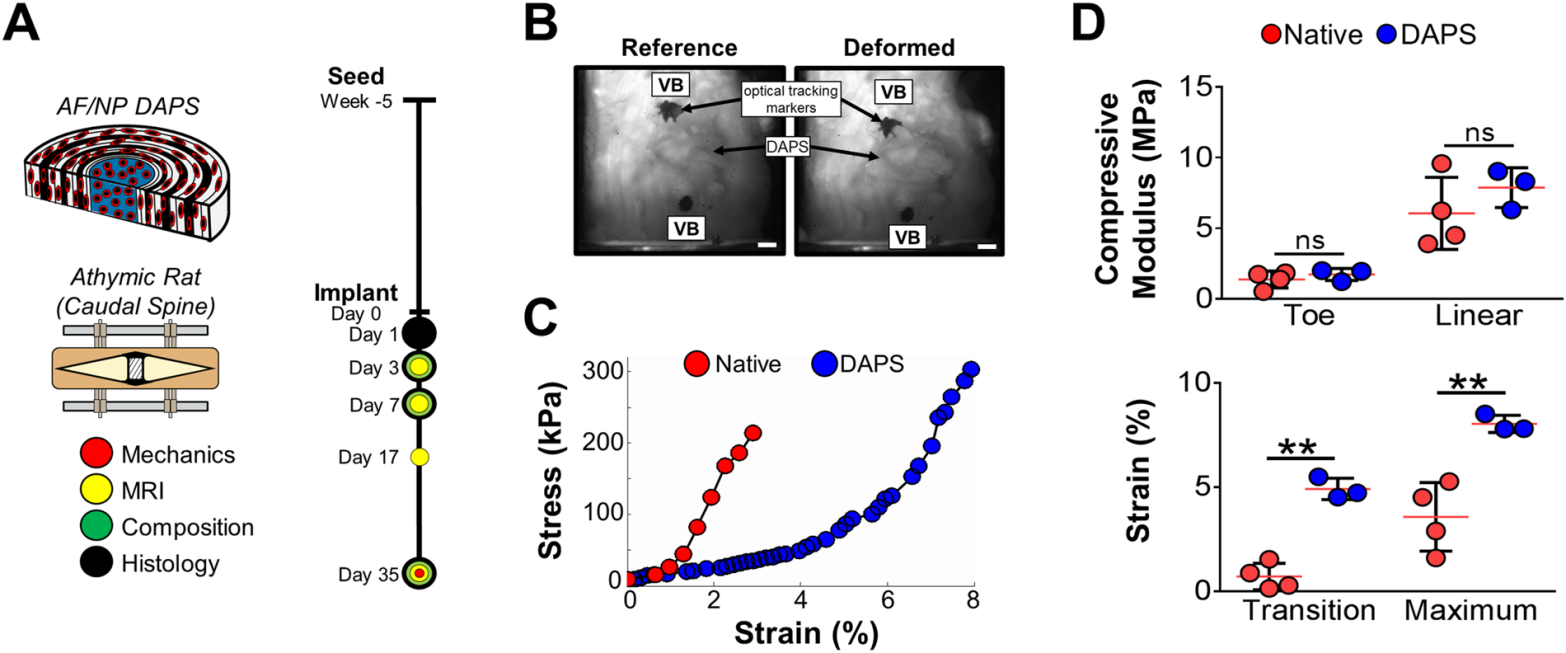
*In Vivo* DAPS Implantation: Immature constructs. **(A)** *Study design:* After 5 weeks of *in vitro* culture, AF/NP DAPS were implanted into the caudal spines of athymic rats using an external fixation device to stabilize the implantation space. **(B)** *Mechanical testing with optical displacement tracking:* After 5 weeks *in vivo*, vertebra-DAPS-vertebra segments were tested in compression using an optical displacement tracking technique to measure deformation. Scale: 1 mm. **(C)** *Representative stress-strain curves:* Similar to native disc segments, DAPS had a nonlinear stress-strain response with distinct toe and linear regions. **(D)** *Mechanical properties:* There were no significant differences between toe and linear region moduli of DAPS constructs and native discs. The primary difference was in the transition and maximum strains, which were higher in the DAPS group. (ns, non-significant; **p < 0.01).

Based on these promising *in vitro* findings, we sought to identify an appropriate pre-culture duration for *in vivo* implantation. To test the hypothesis that immature constructs with a rapid growth rate robustly integrate into native tissue^18^, DAPS were cultured for 5 weeks (an immature state) and implanted into the caudal spines of athymic rats^16,19^ [Fig. 2A]. After 5 weeks *in vivo*, vertebra-DAPS-vertebra segments were isolated and subjected to cyclic compressive loading (20 cycles, from 0 to 3 N, ≈ 0 to 0.3 MPa) [Fig. 2B]. Implanted DAPS were functional at physiologic loads [Fig. 2C], matching native compressive moduli (toe region: 1.7 ± 0.4 MPa; linear region: 7.9 ± 1.4 MPa) [Fig. 2D]. The strain at which DAPS transitioned between low and high-stiffness regions was higher than native tissue (p < 0.05; transition strain: DAPS vs. native) and, consequently, the maximum strain at 3 N was higher (p < 0.05; maximum strain: DAPS vs. native) [Fig. 2D].

**Figure 3:**
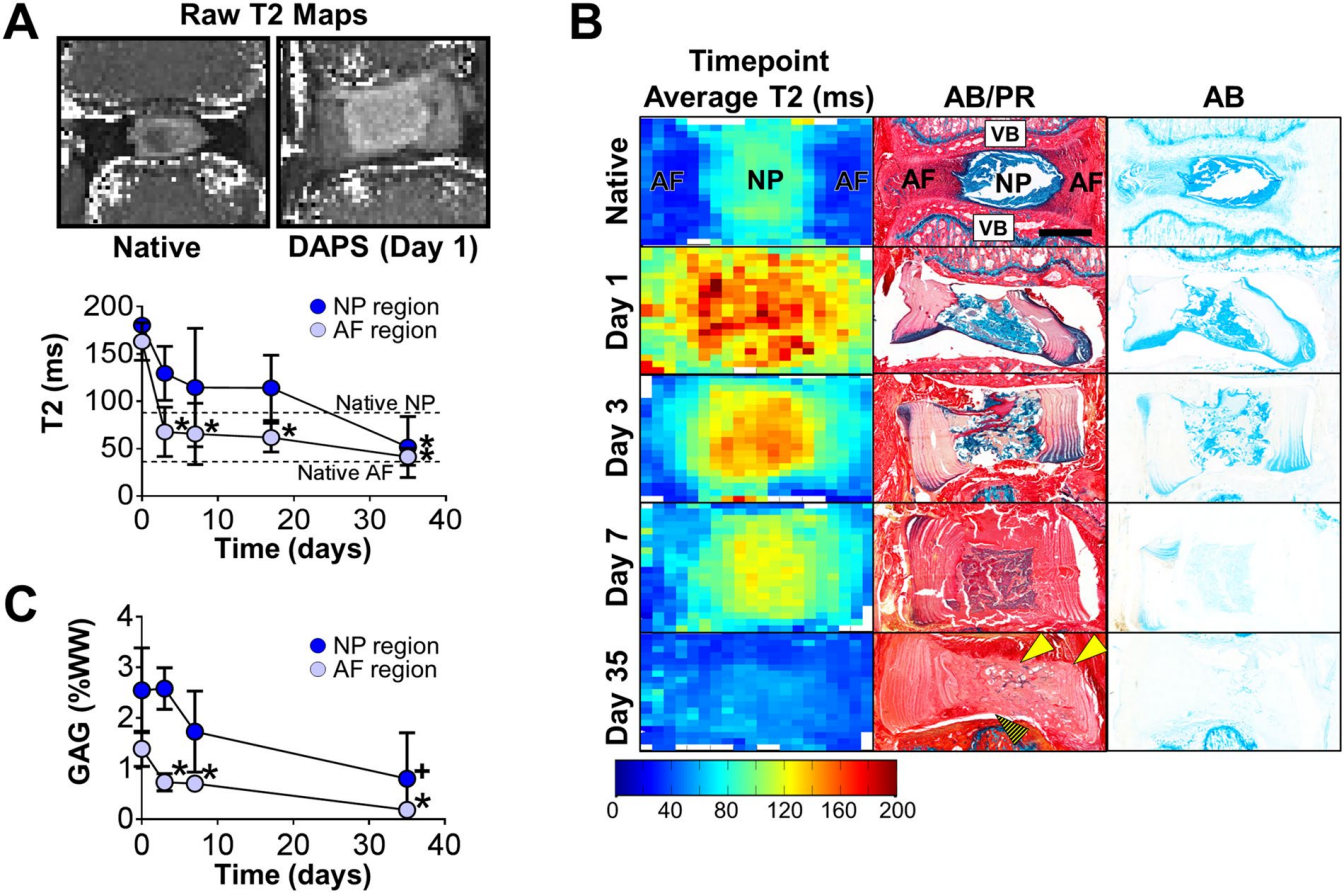
*In Vivo* DAPS Implantation: Immature Constructs (continued). **(A)** *Raw T2 maps and quantification:* T2 MRI mapping was performed over the 5-week implantation period, and the T2 relaxation time (indicative of water and GAG content) was calculated after manual segmentation of the AF and NP regions. Both the AF and NP T2 decreased significantly after implantation (*p < 0.05 vs. day 1). **(B)** *Comparison of MRI and histology:* Time point average T2 maps (left column) were generated and compared to Alcian blue (AB)/picrosirius red (PR) stained histological sections (middle column) and AB-only stained sections (right column). Corresponding with the decrease in T2, AB-positive staining decreased over time in both the AF and NP. Remodeling to the NP region took place over this same time period, with the NP transitioning from a PG-rich to collagen-rich state over 5 weeks. There was evidence of DAPS integration into native soft tissue (solid arrows) but not into adjacent vertebrae (striped arrow). Scale: 1 mm. **(C)** *GAG quantification:* GAG depletion in both the AF and NP regions was confirmed by direct measurement (*p < 0.05 vs. day 1, + p < 0.1 vs. day 1).

To evaluate post-implantation adaptation, T2 MRI maps (indicative of GAG and water content^20^) were acquired at 4.7 T [Fig. 3A, top]. The T2 relaxation times of DAPS AF and NP regions were higher than native tissue at implantation, but progressively decreased over 5 weeks (p < 0.05; AF region: day 1 vs. day 3, day 7, week 2.5, week 5; NP region: day 1 vs. week 5) [Fig. 3A, bottom]; this was confirmed by time point average maps^21^ [Fig. 3B, left column]. Alcian blue staining supported decreased T2 as evidenced by PG depletion in the NP region [Fig. 3B, middle and right columns]. This was further confirmed by direct measurements of NP region GAG (p < 0.05; AF region: day 0 vs. day 3, day 7, day 35; p < 0.1; NP region: day 0 vs. day 35) [Fig. 3C]. After 5 weeks of *in vitro* pre-culture, NP composition was PG- and collagen-rich [Fig. 1C], however, by 5 weeks post-implantation, DAPS were composed primarily of collagen, indicating a shift in composition [Fig. 3B, middle column]. This phenotypic alteration initiated in the NP at days 1 and 3, with degradation of the NP region apparent by day 7; by day 35, the original NP was difficult to distinguish. The AF region retained its structure over time, but was primarily collagen at day 35 as well. Integration of DAPS into the adjacent soft tissue was evident at 5 weeks [Fig. 3B, middle column, solid arrows] while integration of DAPS into adjacent vertebrae was not [Fig. 3B, middle column, striped arrow]. Thus, early functional performance (indicated by compressive mechanical testing) may have been due to DAPS integration into the surrounding soft tissue, rather than the adjacent vertebrae (which would be necessary to withstand multi-axial physiologic loads).

As the NP affects the native mechanical response at low loads^22,23^, we hypothesized that high transition strains relative to the native [Fig. 2D] were driven by NP region dysfunction. Thus, we excised the NP region from 10-week *in vitro* matured DAPS and performed compression tests; confirming this hypothesis, we found that the transition strain increased with NP removal [Fig. S2].

### Implantation of Fully Mature DAPS

**Figure 4:**
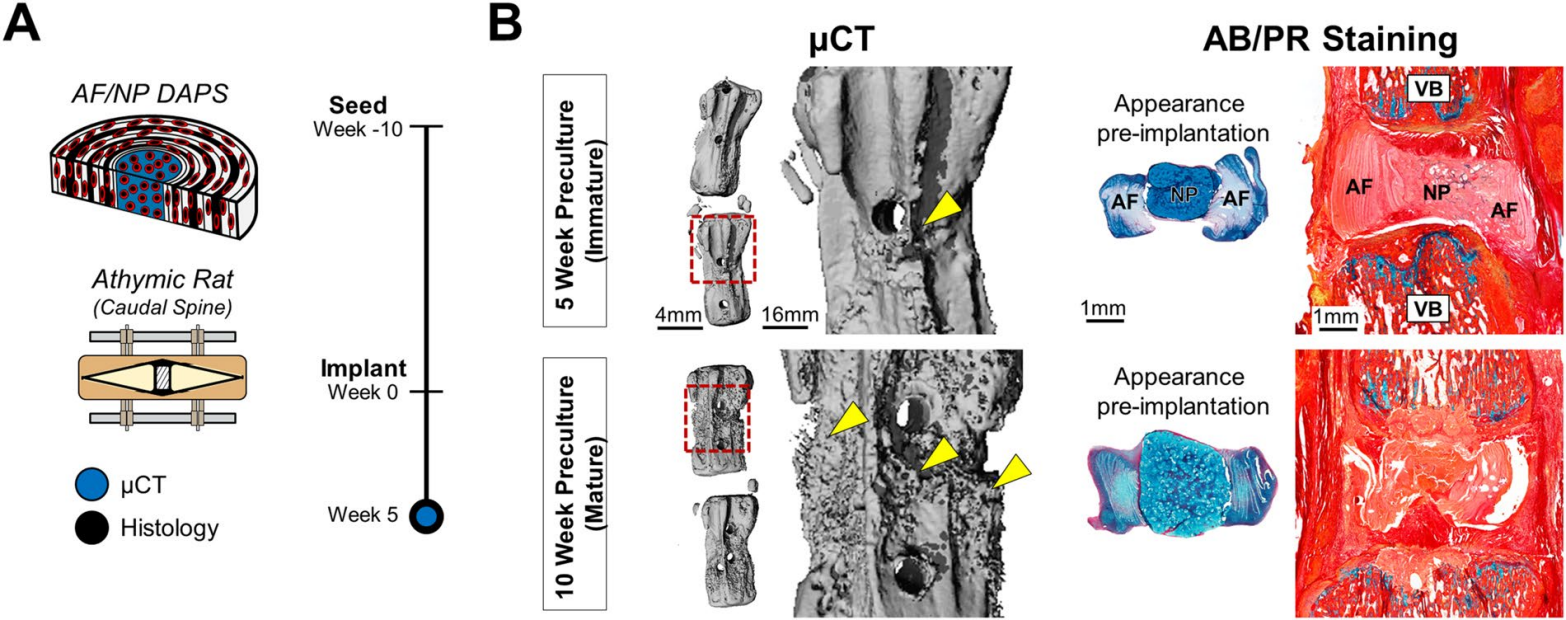
Implantation of Fully Matured DAPS. **(A)** *Study design:* AF/NP DAPS were pre-matured for 10 weeks, implanted into the caudal spines of athymics rats, and analyzed by µCT and histology after 5 weeks. **(B)** *Implantation results:* µCT reconstructions and Alcian blue/picrosirius red stained sections of 10-week pre-cultured DAPS after 5 weeks *in vivo* are displayed along with the 5-week pre-cultured group for side-by-side comparison. There was evidence of bone deposition on the vertebral surface (inset, arrows) but no evidence of fusion. PG-positive staining was not evident in either the AF or NP, and constructs did not integrate into adjacent vertebrae.

Given that immature DAPS did not retain pre-implantation properties or integrate into adjacent vertebrae, we hypothesized that a mature construct may be better suited for the *in vivo* environment. Thus, DAPS were pre-cultured for 10 weeks (a mature state) and implanted into the caudal spines of athymic rats [Fig. 4A]. After 5 weeks, microcomputed tomography (μCT) reconstructions revealed normal vertebral morphology, with bone formation located only at the surgical wire holes [Fig. 4B, arrows] and no bone formation in the intervertebral space. Histological staining of vertebra-DAPS-vertebra sections showed, however, that mature constructs also showed little evidence of NP PG [Fig. 4B] or vertebral integration. Thus, *in vivo* structural and compositional changes to DAPS were independent of maturation state.

### Subcutaneous Implantation of DAPS

**Figure 5:**
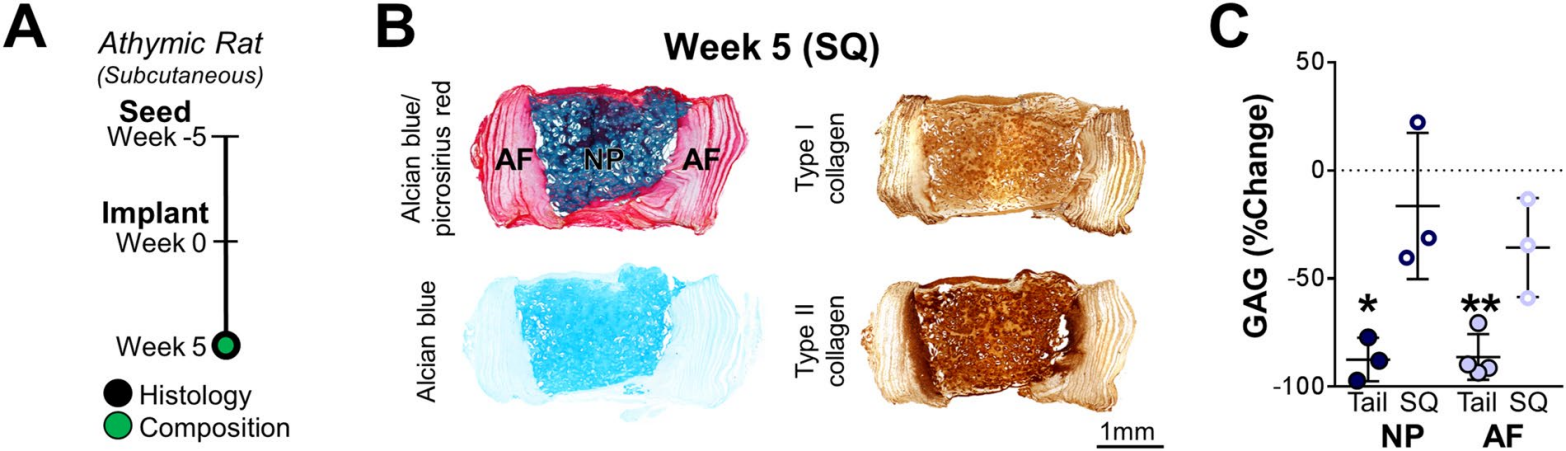
Subcutaneous Implantation of DAPS. **(A)** *Study design:* To compare implantation sites, DAPS were pre-matured for 5 weeks, implanted into the dorsal subcutaneous space of athymic rats, and, after 5 weeks, histological and GAG content analyses were performed. **(B)** *Histological analysis:* There were no gross structural or compositional changes to the NP region. Alcian blue staining remained strongest in the NP region and picrosirius red staining remained strongest in the AF region. Types I and II collagens were abundant in the NP region, and in the interlamellar spaces of the AF. **(C)** *GAG quantification:* Measurements of AF and NP region GAG showed a significant decrease after 5 weeks of implantation into the tail space, while there were no significant decreases with SQ implantation over this same time (*p < 0.05 vs. week 0, **p < 0.001).

We next asked whether this phenotype shift was a consequence of *in vivo* implantation in general (i.e. removing cells from the *in vitro* surplus of nutrients) or due to the intervertebral space specifically. To test this idea, AF/NP DAPS were matured for 5 weeks *in vitro* and then implanted in the subcutaneous (SQ) space of athymic rats [Fig. 5A]. Contrary to our findings for *in situ* constructs, there was no change in GAG in either the AF or NP regions (p < 0.05; AF region, tail: week 0 vs. week 5; NP region, tail: week 0 vs. week 5) [Fig. 5B,C]. The NP structure remained intact and retained types I and II collagen, while the AF region retained interlamellar collagens and PG [Fig. 5B]. This suggested that the *in vivo* disc environment (with invasive surgery, intraoperative bleeding, postoperative edema) presented a challenge for phenotype retention, particularly in the NP region. Conversely, the SQ environment (with minimally invasive surgery, minor bleeding) was amenable to implant survival.

### Culture and Implantation of DAPS with Engineered Endplates (eDAPS)

**Figure 6:**
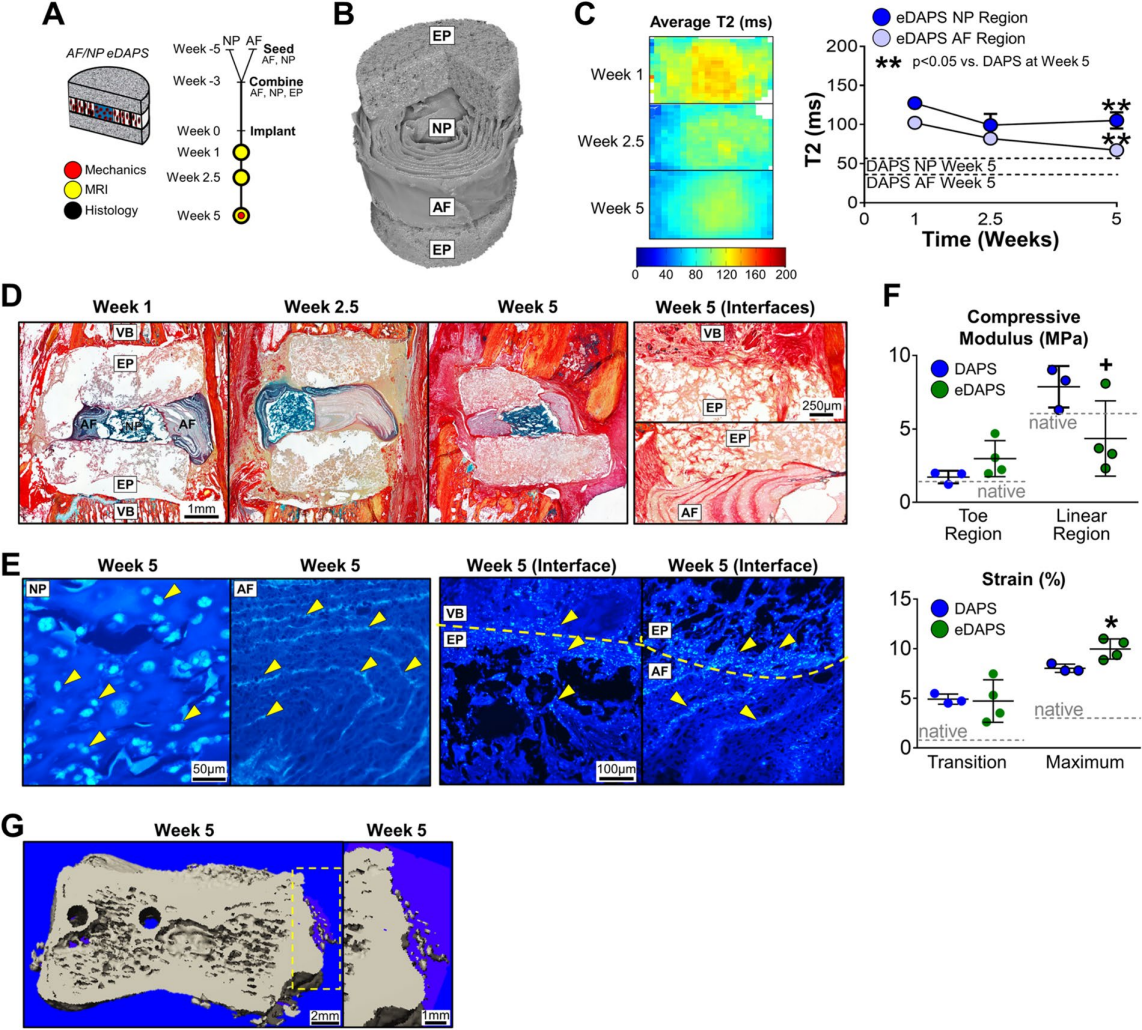
Culture and Implantation of DAPS with Engineered Endplates (eDAPS). Given the evidence of poor integration seen in the above studies, DAPS were combined with porous polymer endplates to evaluate whether endplates could bridge the DAPS/vertebra interface and improve tissue integration. The following acronyms are used: EP: endplate, NP: nucleus pulposus, AF: annulus fibrosus, VB: vertebral body. **(A)** *Study design:* AF and NP regions were separately seeded and cultured, and then combined along with the acellular endplates to form eDAPS. eDAPS were implanted into the tails of athymic rats and evaluated by MRI, histology, and mechanical testing over 5 weeks *in vivo*. **(B)** *µCT:* 3D µCT reconstruction of an acellular eDAPS, with cut-away illustrating the lamellar AF structure and porous endplate. **(C)** *MRI:* (left) Population average T2 maps of DAPS after 1, 2.5, and 5 weeks of implantation. (right) Mean T2 relaxation time of eDAPS AF and NP regions. eDAPS maintained a higher T2 relaxation time 5 weeks after implantation. (**p < 0.001 vs. DAPS at that time point). **(D)** *Histological analysis:* Alcian blue/picrosirius red-stained sections of DAPS after implantation. The endplate regions gradually filled with tissue over 5 weeks, with positive collagen staining throughout, while the AF and NP regions maintained PG positive staining over the 5-week implantation period. (D, ‘Interfaces’ top) The endplate/vertebra boundary was bridged with new collagenous tissue that had formed after removal of the vertebral endplate. (D, ‘Interfaces’ bottom) The AF/endplate interface was also bridged by collagenous tissue that was contiguous with tissue within the AF interlamellar spaces. **(E)** *Staining for Cell Nuclei:* DAPI staining of the AF, NP, and AF/endplate and vertebra/endplate interfaces. The NP region was filled with rounded cell nuclei typical of cells cast in a hydrogel, while the AF region was filled with elongated cell nuclei in the interlamellar spaces. (E, ‘Interface’ left) The vertebra/endplate interface showed evidence of cells in both regions, suggesting that cells from the vertebrae had crossed the interface into the endplate. (E, ‘Interface’ right) Similarly, The AF/endplate interface showed evidence of cells in both regions, where AF cells had migrated into the endplate region. **(F)** *Mechanical function:* Mechanical properties of eDAPS were similar to DAPS at lower strains (toe region modulus, transition strains). At higher strains, eDAPS were less stiff than DAPS without endplates (linear region modulus, maximum strain) though still mechanically robust and comparable to native discs (linear region modulus = 4.4 ± 2.6 MPa). (native rat caudal disc values are shown as dotted lines, + p < 0.1 vs. DAPS; *p < 0.05 vs. DAPS). **(G)** *µCT:* There was evidence of early ossification in the eDAPS engineered endplate region.

Given the loss of NP phenotype (coincident with the invasion of host tissue seen histologically), we next considered methods to establish mechanical continuity across the vertebra and isolate the NP region from *in vivo* factors. Building on work from rat femur segmental defect models^24,25^ and composite engineered tissues^26,27^, we designed engineered PCL foam endplates to allow bony ingrowth from adjacent vertebrae and encase/protect the interior DAPS regions. Endplates were cultured in apposition with AF/NP DAPS (held intact with two 31 G needles passed through the AF and endplates from top to bottom) prior to implantation into the rat tail [Fig. 6A] and, after 5-weeks *in vitro*, needles were removed and eDAPS [Fig. 6B] were implanted into the rat tail, using a pneumatic burr to remove the native vertebral endplates and expose a trabecular surface for integration.

Endplates had a protective effect on both the NP and AF regions, mitigating the loss of PG that occurred previously. This was confirmed by T2 MRI, where the NP T2 relaxation time remained high and was significantly greater than in DAPS without endplates at 5 weeks (p < 0.05, eDAPS AF region vs. DAPS AF region at Week 5 and eDAPS NP region vs. DAPS NP region at Week 5) [Fig. 6C]. Histologically, Alcian blue positive staining was apparent in the NP, further suggestive of high PG content at each time point [Fig. 6D], and integration at both the eDAPS/endplate interface and the endplate/vertebra interface was apparent after 5 weeks [Fig. 6D, right]. At the endplate/vertebra interface, new collagenous tissue formed in areas where the original vertebral endplate was located, and this tissue infiltrated into and through the engineered endplate. At the AF/endplate boundary, a collagen-rich interface developed that was contiguous with tissue formed in the AF interlamellar spaces. Cells continued to populate the AF and NP regions of the construct after 5 weeks *in vivo*, while cells from the DAPS AF region and adjacent vertebrae had migrated into the endplate regions [Figs 6E, S3]. eDAPS also retained their mechanical integrity, with a linear region compressive modulus that was near native levels [Figs 6F, S4].

## Discussion

Total disc arthroplasty is a promising solution to maintain spine function in the advanced stages of disc disease. Current metal-on-plastic prosthetic discs aim to restore the native spine kinematics, but are subject to subsidence- and migration-related complications, as their constituent materials do not have physical properties that match native tissue. In this study, we demonstrated that key features of spine function can be replicated *in vivo* by replacing the native disc with a biologic substitute. We applied physiologic loading and found that segments containing implanted DAPS closely approximated the native disc compressive modulus. Furthermore, DAPS did not instigate intervertebral fusion and, with the inclusion of engineered endplates, integrated into native vertebrae, maintained pre-implantation, composition and contained cells. These data support the DAPS technology for future clinical translation.

With extended *in vitro* growth, compositional and functional properties of DAPS reached levels comparable to native human and rat disc. While the *in vitro* elastic (time-***in***dependent) mechanical properties of DAPS were generally unaltered over time in culture, the development of viscoelastic (time-***de***pendent) mechanical properties, along with improvements in extracellular matrix content, demonstrated the *in vitro* transition from a cell-seeded biomaterial to a hydrated, engineered disc-like tissue. Despite increasing extracellular matrix content over time *in vitro*, the compressive mechanical properties did not change. Electrospun PCL has a tensile modulus in the MPa range^28^ and slowly degrades^29^; PCL is likely the dominant factor in the elastic mechanical response. Additionally, in parallel with increasing GAG content and size (swelling) *in vitro*, DAPS mechanical properties became increasingly hydration-dependent, as evidenced by increased creep strain and model parameters. The stability of DAPS constituent materials and resultant elastic mechanical behavior, as well as DAPS durability through 5,000 cycles of fatigue loading, likely contributed to the robust mechanical response maintained after implantation.

In the *in vitro* model, TGF-β promoted a strong chondrogenic phenotype, driving seeded cells to produce types I and II collagen and proteoglycans, rapidly imparting functional stability to the engineered constructs. The intervertebral disc contains types I and II collagen in specific distribution pattern; type I collagen is highly concentrated in the peripheral annulus and decreases in concentration moving radially inward towards the nucleus; type II collagen is highly concentrated in the central nucleus and decreases in concentration radially outward. Immunostaining results demonstrated that the distribution of types I and II collagen was symmetric throughout the DAPS after *in vitro* culture, likely due to the uniform chondrogenic signal provided by TGF-β supplementation. However, once implanted, when the powerful TGF-β signal was removed, DAPS were similar in histological appearance to native discs in terms of proteoglycan and types I and II collagen distribution [Figs 5B and 6D]. Previous work in our lab and others has demonstrated that, in terms of cell morphology and matrix deposition, hyaluronic acid hydrogels promote a chondrogenic phenotype^30,31^ while aligned scaffolds produce a fibro-chondrogenic phenotype^32,33^. It may be that the scaffold is more important for driving cell behavior following *in vivo* implantation than supplementation with phenotype-influencing growth factors during the pre-culture period.

DAPS replicated aspects of native disc function *in vivo*, achieving nonlinear stress/strain behavior and compressive moduli matching the native rat disc, an improvement over previous implants^15,34,35^. Without an endplate to interface with adjacent vertebrae, fibrous tissue formed at the DAPS/vertebra boundary, the NP region degraded, and there was an increased transition strain. This interface likely contributes to the functional response at low loads and the disorganized tissue (and deficient NP) may contribute to the extended toe region. In support of this, after excising the NP region, the toe region elongated, suggesting that the engineered NP region contributes to the mechanical function of DAPS at low loads, mimicking native disc function^22,23^.

Engineered endplates improved integration into adjacent vertebrae and eliminated the fibrous tissue at the DAPS/vertebra interfaces. Implanted eDAPs retained a high level of PG content in the NP region (compared to DAPS without an endplate and native tissue [Fig. S4]) and segments with an eDAPS supported physiologic loads (~1X rat body weight). There was evidence of collagen formation and mineral deposition in the engineered endplate region [Fig. 6G]. With additional time *in vivo* and the resumption of physiological loading, the engineered endplates may fully ossify, improving integration and segmental rigidity, while also serving as a physical barrier to factors that affected NP composition. Similar PCL foams succesfully bridged segmental defects in rat femurs, and can be modified with minerals or growth factors to expedite integration and ossification^24,25^; future work will evaluate integration strength at these interfaces. Our working strategy is to provide a period of immobilization immediately postoperatively, allowing for integration in a stable environment, followed by the remobilization and the resumption of normal loading and range of motion. Temporary immobilization may not be necessary for the lumbar spine, which has limited range of motion compared to the very flexible rat tail, but could be achieved clinically through a prescribed period of bracing following surgery. Data on the time-course of integration strength will determine the length of the immobilization period.

DAPS cultured *in vitro* met or exceeded many compositional and mechanical design criteria for engineered discs. Despite this, without endplates and upon exposure to the rat tail environment, there was a significant loss of PG. In other studies, this transition from the amenable *in vitro* culture environment to the harsh *in vivo* environment has also had a negative effect; in many cases, engineered tissues do not retain their pre-implantation phenotype after short periods *in vivo* ^36–39^. This shift in phenotype may be the consequence of direct exposure to blood at the implant site, as NP degradation was muted following SQ implantation (where intraoperative bleeding is minimal). Exposure to blood and serum negatively affects cell behavior; when cartilage is exposed to whole blood, there is decreased PG synthesis and increased chondrocyte apoptosis^40^. Furthermore, blood products may have also affected the HA hydrogel; rapid HA degradation was apparent in both a rat tail model^17^ and a pig osteochondral defect model^41^. Conversely, in other SQ models, HA remained intact^42,43^. NP PG loss was attenuated via the inclusion of an engineered endplate, which may act as a sink for native blood cells or inflammatory signals that would otherwise invade the construct and affect DAPS composition; further examination of this phenomenon is required. New strategies for transitioning engineered tissues directly exposed to blood or other body fluids, such as engineered cartilage (synovial fluid), may be required for sustained function. Furthermore, the ultimate goal for this technology is to replace a severely diseased disc when the local microenvironment may have diminished vascular supply, endplate calcification, inflammation, and abnormal spine function. Ultimately, these engineered replacement discs must be evaluated in *in vivo* models of degenerative disc disease to fully assess their ability to restore function in this challenging context.

In this study, we focused on the sustainability of the implant on a macroscopic level and overcame issues related to implant integration and degradation. Having improved this model of total disc replacement, future work will further evaluate implant viability on the cellular level. In previous work, we demonstrated that cell metabolic activity in both the DAPS AF and NP regions was maintained following subcutaneous implantation^44^, and in the current study, we demonstrate that cells are present in the eDAPS following 5 weeks of implantation. Tracking implant cellularity in the rat tail model is confounded by the presence of host cells invading the engineered disc^17^. Future work must evaluate cell behavior in DAPS pre- and post-implantation more closely, differentiate between host and implant cells, characterize implanted cell behavior, and investigate clinically-relevant cell populations. Furthermore, cell viability and metabolism may be also compromised when scaling rat-sized implants to larger dimensions as nutritional pathways increase in length. For *in vitro* pre-culture, including nutrient channels is one method to improve the availability of nutrients^45,46^ and bioreactors can improve diffusion, while also promoting matrix elaboration through mechanobiological signaling^47,48^. These technologies may hasten the *in vitro* growth and minimize pre-culture periods. For *in vivo* implantation, nutrient availability will be affected by the engineered endplate thickness and porosity, the local mechanical environment (where a mobilized segment may show improved potential for nutrient convection^49^), and the ability to restore vascularity by removing a calcified or degenerated endplate and accessing the subchondral blood supply. Ongoing studies are addressing these issues as we translate this technology to larger animal models.

To conclude, the intervertebral discs are implicated in low back pain as their degeneration leads to the deficits in spine function. Tissue engineering is one strategy to replace severely degenerated discs and, in these preclinical studies, a biologic replacement disc integrated into the spine, maintained cell viability and composition, and replicated aspects of healthy disc function after long-term implantation. Importantly, our findings show that standard *in vitro* culture conditions may not produce an engineered tissue that is sustainable after the transition to the challenging *in vivo* environment. Here, engineered endplates shielded the construct from the *in vivo* environment and prevented NP remodeling. These findings strongly support the DAPS technology as a translatable strategy for engineered total disc replacement, with future studies in large animal models as the next step toward clinical translation.

## Materials and Methods

### Cell Isolation and Expansion

MSCs were isolated from femoral and tibial bone marrow of juvenile cows (~2–3 months old and ~15 hours after sacrifice; Research 87 Inc., Boylston, MA), while AF and NP cells were isolated from the caudal discs (~3 years old and ~2 hours after sacrifice; JBS Souderton Inc., Souderton, PA)^14,50^. In preparation for seeding, all cell types were expanded to passage 2 or 3 in a basal medium (BM) that included Dulbecco’s Modified Eagle Medium (DMEM; Gibco, Invitrogen Life Sciences, Carlsbad, CA), 10% fetal bovine serum (Gibco) and 1% Antibiotic-Antimycotic (Gibco). We received approval for isolating bone marrow and caudal discs cells from the Corporal Michael J. Crescenz Veterans’ Affairs Medical Center Institutional Animal Care and Use Committee (IACUC) and followed all guidelines dictated by the committee in performing these experiments.

### DAPS Fabrication, Cell Seeding, and *In Vitro* Culture

The AF portions of DAPS were fabricated from an electrospun nanofibrous scaffold to match the rat caudal disc geometry^15,16^. Layers of poly(ε-caprolactone) (PCL) nanofibers and poly(ethylene oxide) (PEO) were sequentially electrospun onto a rotating mandrel as aligned multilayer sheets. This scaffold was cut at an angle into strips in which fibers ran 30° to the strip length, replicating the structure of an individual lamella in the native AF. Strips with alternating alignment were wrapped around a post into concentric discs with final dimensions 4–5 mm diameter and 2 mm height. Scaffolds were hydrated and sterilized through a series of gradient ethanol washes, which removed water-soluble PEO layers, and then were coated with a 20 μg/mL fibronectin solution (Sigma-Aldrich, St. Louis, MO) to improve cell attachment^32^. Bovine MSCs or AF cells were seeded on the top and bottom side of the AF regions (2 × 10^6^ cells per construct), allowing 1 hour of cell attachment per side.

The NP portions of the DAPS constructs were generated using a photo-crosslinkable bioactive hydrogel^31,51^. Methacrylated hyaluronic acid (MeHA) was produced by reacting 65 kDA sodium hyaluronate (Lifecore, Chaska, MN) with methacrylic anhydride (Sigma Aldrich, St. Louis, MO) as in^51^. MeHA was sterilized by 15 minutes of UV light exposure, after which 0.05% photoinitiator (Irgacure 2959, (2-methyl-1-[4-(hydroxyethoxy)phenyl]-2-methyl-1-propanone, Ciba-Geigy, Tarrytown, NY) was added for make the 1% w/v solution of MeHA. Bovine MSCS or NP cells were suspended in the MeHA solution (20 M cells/mL, 6 × 10^5^ cells per construct), which was poured into a mold and photo-polymerized with UV light exposure for 10 minutes. The mold was punched to create cell-laden cylindrical gels of final dimensions 2 mm diameter by 1.5 mm height.

Following fabrication and seeding, AF and NP regions were cultured separately for 2 weeks, at which point a 2 mm core was punched from the center of the AF, and the cell-seeded NP was inserted to form the DAPS construct. DAPS were cultured in a chemically defined medium^52^ consisting of DMEM supplemented with 1% penicillin, streptomycin, and amphotericin B (Antibiotic-Antimycotic; Gibco), 40 ng/mL dexamethasone (Sigma-Aldrich), 50 μg/mL ascorbate 2-phosphate (Sigma-Aldrich), 40 μg/mL L-proline (Sigma-Aldrich), 100 μg/mL sodium pyruvate (Corning Life Sciences, Corning, NY), 0.1% insulin, transferrin, and selenious acid (ITS Premix Universal Culture Supplement; Corning), 1.25 mg/mL bovine serum albumin (Sigma-Aldrich), 5.35 μg/mL linoleic acid (Sigma-Aldrich), and 10 ng/mL TGF-β3 (R&D Systems, Minneapolis, MN). This media was refreshed every 3 days.

### Geometric and Mechanical Characterization of DAPS Cultured *In Vitro*

DAPS geometry and compressive mechanical properties were measured at each *in vitro* time point (n = 4/cell type/time point). DAPS were measured for total cross-sectional area by analyzing digital images with a custom MATLAB program^16,53^. Mechanical properties were evaluated in unconfined compression on an electromechanical testing system (5542; Instron, Norwood, MA) with a protocol used to assay native tissue^4,54^. First, a 0.05 N preload was applied and then 20 cycles of compressive loading from 0.05 N to 3.0 N were applied at a rate of 0.5 Hz. This was followed by a return to 0.05 N, and then, to characterize creep response, a 1.5 second step load to 3.0 N was applied, with this load held for 10 minutes while measuring tissue displacement.

AF/NP DAPS were also tested in compression through 5000 cycles to determine their fatigue behavior. DAPS were loaded in compression from 0.05 to 3.0 N at a rate of 0.5 Hz. The strain was recorded at peak load in each cycle. At 20, 1000, and 5000 cycles, a secondary test was conducted at 0.05 Hz to evaluate the linear region compression modulus (n = 3/cycle count).

AF/NP DAPS were also tested without the NP region in order to evaluate the contribution of the NP to the overall mechanical response (n = 3). To do so, the NP region was removed using a 2 mm biopsy punch, and the remaining AF-only construct was tested in cyclic compression as described above.

### Compositional and Histological Evaluation of DAPS

Following mechanical testing, AF and NP regions were separated, digested in proteinase K at 60 °C, and total glycosaminoglycan (GAG) and collagen contents were measured for each region (n = 4/cell type/time point). GAG content was evaluated using the dimethylmethylene blue (DMMB) assay^55^, and collagen content (following acid hydrolysis) using the p-diaminobenzaldehyde/chloramine-T technique for hydroxyproline^56^. Results are reported as normalized to AF or NP region wet weight.

An additional group of DAPS without prior mechanical testing was used for histological analysis (n = 2/cell type/time point). These were prepared by fixation in formalin at room temperature and embedding in paraffin at 55 °C (below the melting temperature of PCL). Sections were cut to 10 µm, stained with Alcian blue (PG) or picrosirius red (collagen) and imaged in bright-field. Immunohistochemical staining for types I and II collagen was performed by first incubating sections in proteinase K and the blocking with 10% normal goat serum (NGS). Primary antibodies (type I collagen: MAB3391, Milipore, Billerica, MA; type II collagen: II-II6B3, DSHB, lowa City, IA) were applied overnight at a concentration of 10 µg/mL at 4 °C, followed by a 10 minute incubation with secondary antibodies (prediluted reagent: 21537, Millipore) at room temperature. Staining was visualized using the DAB chromogen reagent (DAB 150, Millipore) and imaged in brightfield.

### DAPS Implantation

DAPS were implanted into the caudal spines of athymic rats (Foxn1^rnu^ retired breeders, 465 ± 41 g; Harlan Laboratories, Inc., Indianapolis, IN) using an external fixator designed to unload and stabilize the rat caudal disc space^16^. To do so, two surgical wires were passed laterally through both the C8 and C9 vertebrae and the rigid external fixator was fastened to the wires defining the intervertebral distance. A dorsal skin incision was made, the native C8/C9 disc was removed, and DAPS were implanted directly into the disc space with no further manipulation of the fixator. After surgery, rats were returned to normal cage activity. We received approval to implant DAPS into rat caudal spines from the Corporal Michael J. Crescenz Veterans’ Affairs Medical Center IACUC and followed all guidelines dictated by the committee in performing these experiments.

### Mechanical Testing of Implanted DAPS

Vertebra-DAPS-vertebra segments (after 5 weeks implantation) and native disc segments were tested in compression using an electromechanical testing system (5948; Instron, Norwood, MA) and an optical displacement tracking technique (n = 4/group). In preparation for testing, the skin surrounding the segment was carefully removed (with adjacent muscle and tendon left intact). The ventral bony surface adjacent to the disc space was cleared of soft tissue using a micro-curette and ink spots were drawn onto the vertebral bone surfaces proximal and distal to the disc space to serve as markers for optical tracking. A digital camera (acA3800-14 um; Basler AG, Ahrensburg, Germany) fit with a close-focusing macro video lens (Zoom 7000; Navitar Inc., Rochester, NY) and custom software were used to record motion during testing. The mechanical testing protocol consisted of 20 cycles of compression from 0 to −3N at 0.05 Hz, with images of the segment recorded at 10 Hz. Texture tracking was performed in MATLAB^4^ and the 20^th^ cycle of stress-strain was analyzed to determine segment mechanical properties. These mechanical properties were normalized to cross-sectional area measurements from digital images and disc height from MR images, as described previously^54^.

### Mechanical Data Analysis

The 20^th^ cycle of the load/displacement curve was analyzed for toe and linear region modulus, transition strain, and total compressive range of motion (ROM). First, load and displacement were converted to stress and strain by dividing by the total cross-sectional area and height, respectively. Then, toe and linear region stiffness were calculated using a bilinear fit routine in MATLAB.

The creep response was fit to a 5 parameter viscoelastic constitutive model^4^ [Eq. 1] that defines the compressive strain (ε) and stress (σ) as a function of time (t) and includes an early damping modulus and time constant (E_1_ and τ_1_), a late damping modulus and time constant (E_2_ and τ_2_), and an instantaneous damping modulus (E_3_).

1 $${\epsilon }(t)=\frac{\sigma }{{E}_{1}}(1-{e}^{\frac{-t}{{\tau }_{1}}})+\frac{\sigma }{{E}_{2}}(1-{e}^{\frac{-t}{{\tau }_{2}}})+{E}_{3}$$

Creep strain, or the total change in strain from 1.5 s to 10 minutes, was also calculated.

### µCT and Histological Analysis of Implanted DAPS

Vertebra-DAPS-vertebra segments were imaged by µCT (vivaCT 75; SCANCO Medical AG, Bruttisellen, Switzerland) and evaluated histologically (immature DAPS: n = 6/group/time point, mature DAPS: n = 6). Segments were scanned at an isotropic 20.5 µm resolution to evaluate the appearance of the vertebral bodies and to scan for potential bony fusion across the disc space. Following scanning, segments were fixed in formalin, decalcified in formic acid and embedded in paraffin at 55 °C as above. Sections were cut to 10 µm and stained with both Alcian blue and picrosirius red, and imaged in brightfield.

### MRI Analysis of Implanted DAPS

MRI was performed at 4.7 T (Magnex Scientific Limited, Abington, UK) on DAPS 5 weeks after implantation as well as native rat tail discs (n=3–6/group/time point). A multi-echo-multi-spin sequence was used to acquire quantitative T2 maps (three 0.5 mm thick slices, 16 echoes, TE/TR = 7.84 ms/2,000 ms, FOV = 15 × 15 mm^2^, matrix = 128 × 128, 4 averages). Time point average T2 maps were generated by scaling T2 maps to a normalized grid and averaging the T2 signals of each disc within a time point^21^. Mean NP T2 signals for individual discs were calculated after manual segmentation of the NP.

### Subcutaneous Implantation of DAPS

DAPS were implanted into the dorsal subcutaneous space of athymic retired breeder rats (Hsd:RH-Foxn1^rnu^, male, 9–10 months, 463 g ± 45 g; Harlan Laboratories, Branchburg, NJ) (n = 4). Incisions were made 1 cm lateral to the spine and a subcutaneous pocket was opened. DAPS were inserted into the pocket which was then closed with suture. These implants were evaluated histologically by staining with Alcian blue/picrosirius red, Alcian blue alone, types I and II collagen, and GAG content was measured via the DMMB assay. We received approval for subcutaneous DAPS implantations from the Corporal Michael J. Crescenz Veterans’ Affairs Medical Center IACUC and followed all guidelines dictated by the committee in performing these experiments.

### Fabrication and Implantation of DAPS with Engineered Endplates (eDAPS)

Porous PCL foam endplates were produced using a salt-leaching technique. PCL was dissolved in chloroform (20% w/v) and NaCl (Sigma Aldrich) was sieved to yield a range of particles <106 µm diameter, defining the pore size. These particles were then mixed into the PCL solution (PCL/NaCl mass ratio of 1:4). The mixture was poured into a mold and the solvent was allowed to evaporate for 48 h. The resultant sheet was 1.5 mm in height and individual plugs were removed using a 4 mm biopsy punch for the outer diameter, similar to the DAPS geometry. NaCl was removed through gentle agitation of the punched samples in distilled water for 48 h followed by lyophilization. These constructs were evaluated in compression using the methods described for DAPS above (linear region modulus, 1.2 MPa).

These porous PCL EPs were attached to the top and bottom surfaces of cultured DAPS to form eDAPS, and these constructs were then implanted into the rat caudal disc space. To do so, the AF and NP regions of DAPS were first seeded and cultured separately for two weeks as described above. The cellular AF and NP regions and the acellular endplate regions were then combined and held in apposition by passing two 31 G needles through the AF and endplate regions from top to bottom. These were cultured for an additional 3 weeks and then (after removing needles) implanted into the tails of athymic retired breeder rats (n = 4/time point). µCT imaging of an acellular eDAPS was performed at an isotropic voxel size of 2 µm (µCT 50; SCANCO Medical) to demonstrate overall structure. For implantation, the external fixation device was applied and the native disc was removed. A pneumatic burr was used on both the proximal and distal vertebra to remove the cartilage endplates, the vertebral endplates, and the remaining epiphyseal bone just below the growth plate (~3 mm total). At the time of implantation, needles were removed from the eDAPS, then these constructs were inserted into the patent disc space, and the incision was closed with suture. Integration was investigated qualitatively through visual inspection of the endplate/vertebra interface on picrosirius red/alcian blue stained histological sections.

### Statistical Analysis

*In vitro* and *in vivo* DAPS compositional measurements and mechanical properties were compared by one or two-way ANOVA. Post-hoc pairwise analyses were made using the method of Bonferroni (p < 0.05). Comparisons of pre- and post-implantation GAG content in the rat tail and SQ models were made by t-test. Data are displayed as mean ± standard deviation.

## Electronic supplementary material

Supplementary Material
