# Supplementary material for: In Vitro Maturation and In Vivo Integration and Function of an Engineered Cell-Seeded Disc-like Angle Ply Structure (DAPS) for Total Disc Arthroplasty
Source: Sci Rep. 2017 Nov 17;7:15765. doi: 10.1038/s41598-017-15887-4 (PMC5693867; doi:10.1038/s41598-017-15887-4)
Supplement: Supplementary file 1 — Supplementary Material [file 41598_2017_15887_MOESM1_ESM.pdf]

Title: *In Vitro* Maturation and *In Vivo* Integration and Function of an Engineered Cell-Seeded Disc-like Angle Ply Structure (DAPS) for Total Disc Arthroplasty

Authors: J. T. Martin[1,2,3], S. E. Gullbrand[1,2], D. H. Kim[2], K. Ikuta[1,2], C. G. Pfeifer[1,2], B. G. Ashinsky[1,2], L. J. Smith[1,2,4], D. M. Elliott[5], H. E. Smith[1,2,4], R. L. Mauck [1,2,3,6]*

Affiliations:

[1]Translational Musculoskeletal Research Center, Corporal Michael Crescenz VA Medical Center, Philadelphia, PA.

[2]Department of Orthopaedic Surgery, University of Pennsylvania, Philadelphia, PA.

[3]Department of Mechanical Engineering and Applied Mechanics, University of Pennsylvania, Philadelphia, PA.

[4]Department of Neurosurgery, University of Pennsylvania, Philadelphia, PA.

[5]Department of Biomedical Engineering, University of Delaware, Newark, DE.

[6]Department of Bioengineering, University of Pennsylvania, Philadelphia, PA.

Corresponding Author

Robert L. Mauck, Ph.D.
Mary Black Ralston Professor of Orthopaedic Surgery
Professor of Bioengineering
McKay Orthopaedic Research Laboratory
University of Pennsylvania
424 Stemmler Hall
36th Street and Hamilton Walk
Philadelphia, PA 19104-6081
Phone: 215-898-3294
E-mail: lemauck@mail.med.upenn.edu

Supplemental Figure Captions

Figure S1. Geometry and Creep Model Parameters of Cultured DAPS. **(A)** *Geometry:* The height of both AF/NP and MSC/MSC DAPS significantly increased by week 12.5, while there was no change in cross-sectional area (*, $p<0.05$ vs. week 2.5). **(B)** *Mechanical function in creep:* Time constants, τ_1 and τ_2, representing the early and late response to creep loads, continuously increased with time, while damping moduli, E_1 and E_2, decreased over the first 5 weeks, where they remained after 15 weeks. These findings suggest that DAPS become increasingly viscous over time (*$p<0.05$ vs. week 2.5).

Figure S2. Transition strain of AF/NP DAPS after 10 weeks *in vitro* culture and removal of the NP. DAPS transition strain significantly increased after removal of the NP. (t-test: *$p<0.05$ vs. DAPS).

Figure S3. Visualization of cell nuclei at central and interface regions of eDAPS

Figure S4. Direct Comparison of DAPS and eDAPS after Implantation. **(A)** Time point average T2 maps. **(B)** Representative Stress-strain curves. **(C)** Alcian-blue/picrosirius-red stained sections.

Figure S1

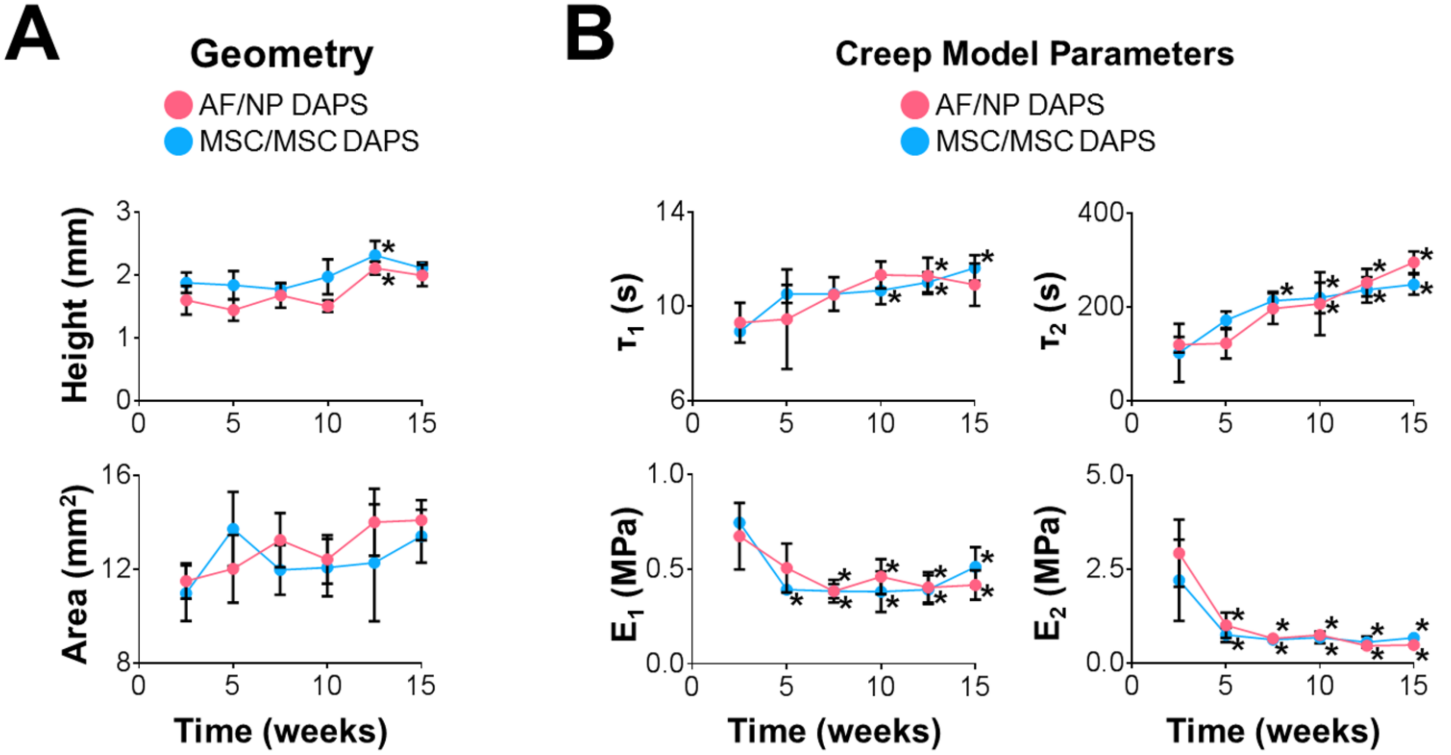

A
Geometry
AF/NP DAPS
MSC/MSC DAPS
Height (mm)
Area (mm²)
Time (weeks)
B
Creep Model Parameters
AF/NP DAPS
MSC/MSC DAPS
τ1 (s)
τ2 (s)
E1 (MPa)
E2 (MPa)
Time (weeks)

Figure S2

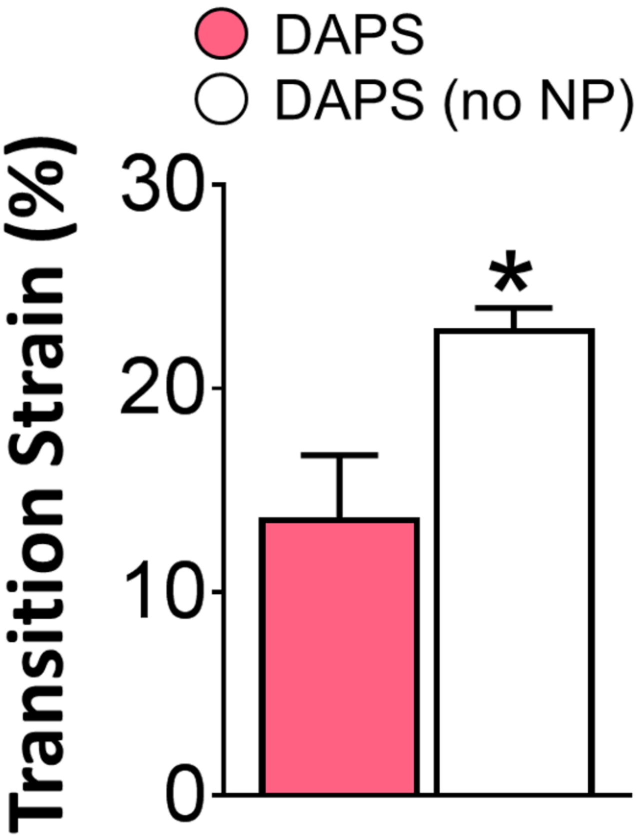

Figure S3

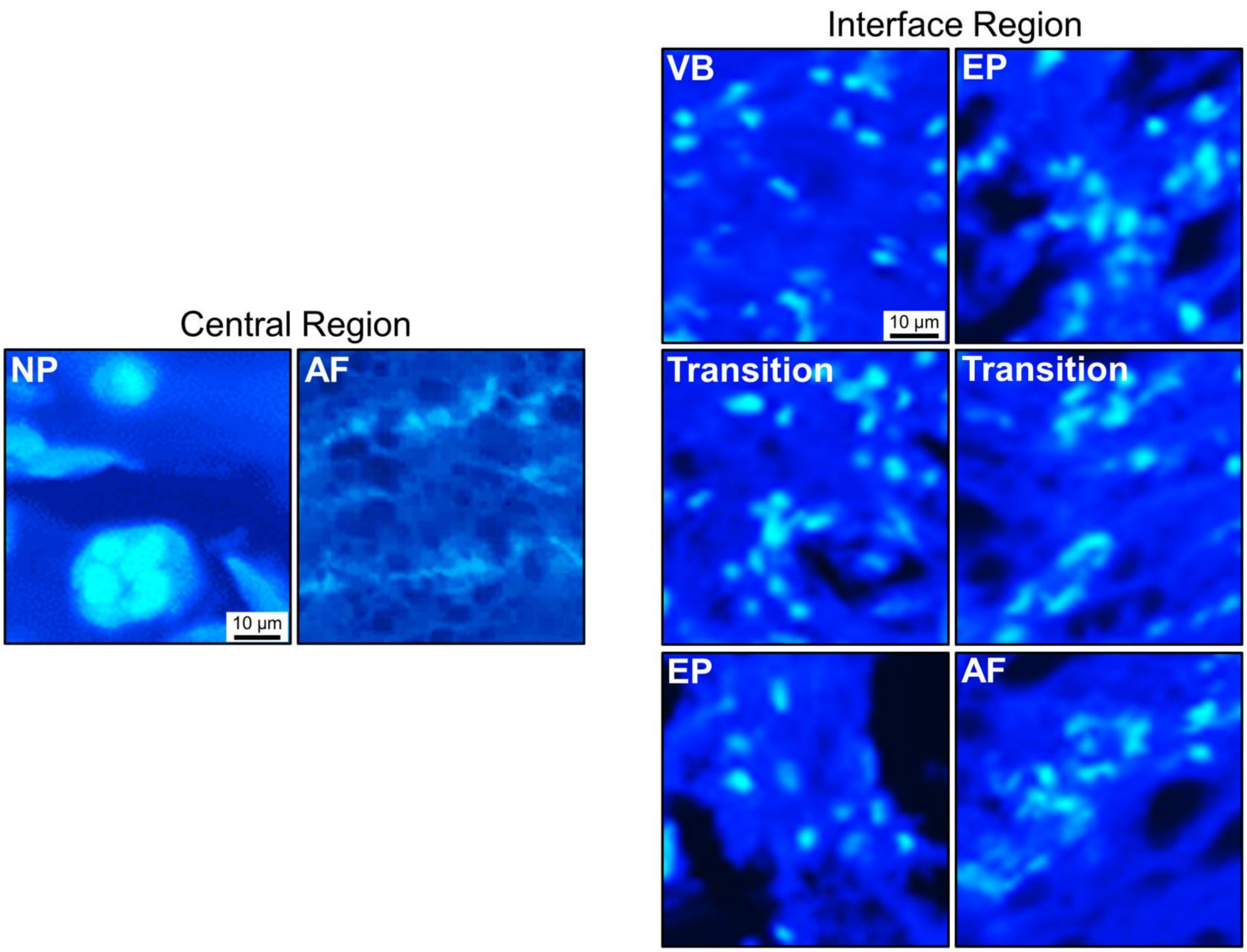
Interface Region
VB
EP
10 µm
Transition
Transition
EP
AF
Central Region
NP
AF
10 µm

Figure S4

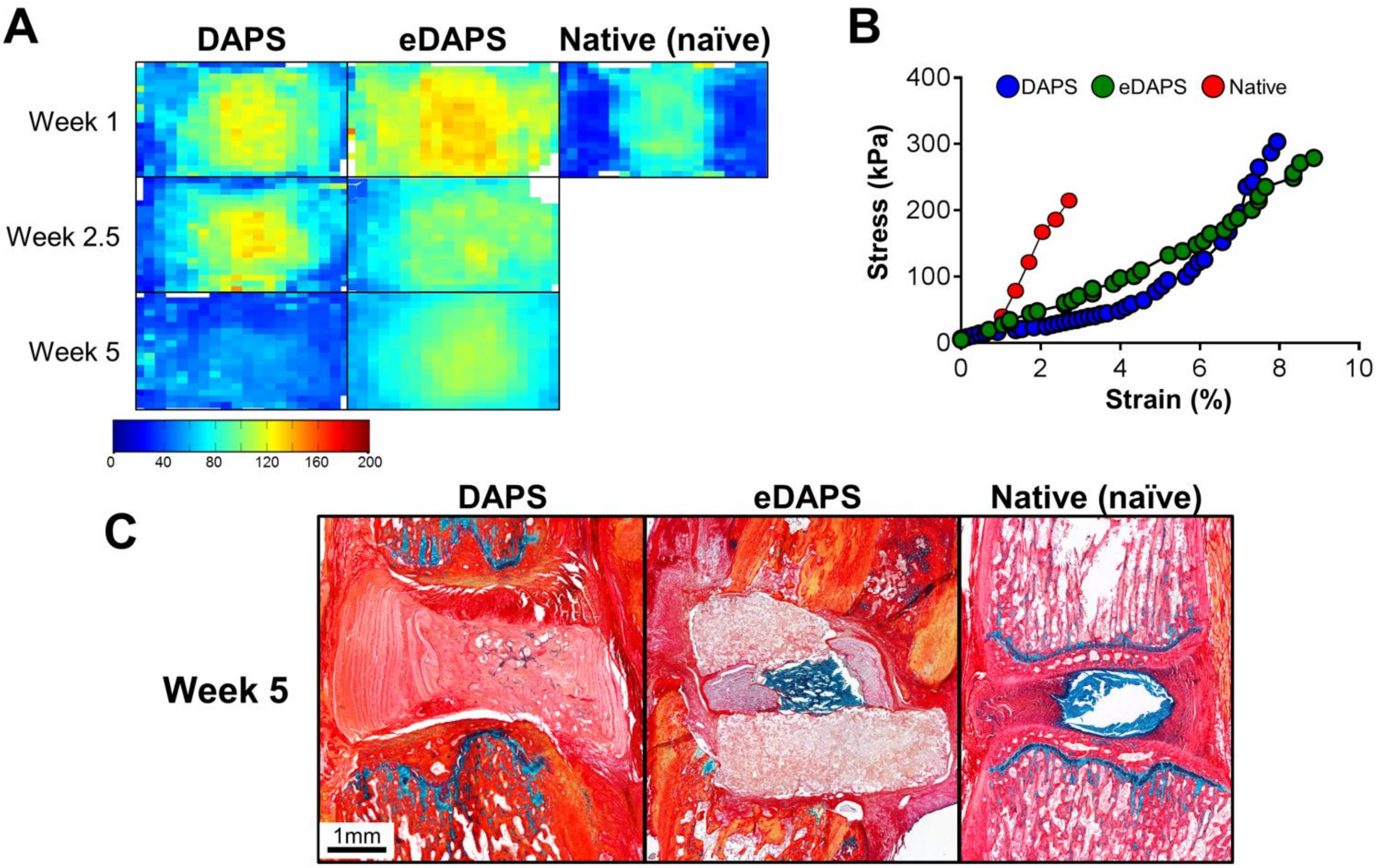
A
DAPS
eDAPS
Native (naïve)
Week 1
Week 2.5
Week 5
0
40
80
120
160
200
B
DAPS
eDAPS
Native
Stress (kPa)
400
300
200
100
0
0
2
4
6
8
10
Strain (%)
C
DAPS
eDAPS
Native (naïve)
Week 5
1mm
